# Mechanical nociceptive thresholds of the foot do not change following anaesthesia of the digital flexor tendon sheath in horses

**DOI:** 10.1002/vro2.70051

**Published:** 2026-11-10

**Authors:** Letícia de Oliveira Cota, Bruno Dondoni Malacarne, Lucas Antunes Dias, Antônio Catunda Pinho Neto, Mayara Gonçalves Correa, Marina Alcantara Cavalcante, Marina Souza Luz de Da Cunha, Camila Ferreira Rodrigues e Silva, Cahuê Francisco Rosa Paz, Armando de Mattos Carvalho, Andressa Batista Silveira

**Affiliations:** ^1^ EQUINOVA Research Group Department of Veterinary Clinic and Surgery Escola de Veterinária Universidade Federal de Minas Gerais Belo Horizonte Minas Gerais Brazil; ^2^ Marion duPont Scott Equine Medical Center/Virginia Tech Leesburg Virginia USA; ^3^ Equine Health and Sports Performances Louisiana State University Baton Rouge Louisiana USA; ^4^ Department of Veterinary Medicine Pontifícia Universidade Católica de Minas Gerais – PUC/MG Belo Horizonte Minas Gerais Brazil; ^5^ Escola de Veterinária Departamento de Clínica e Cirurgia Veterinarias Universidade Federal de Minas Gerais Belo Horizonte Minas Gerais Brazil

**Keywords:** diagnostic analgesia, horse, lameness, lidocaine, pressure algometry

## Abstract

**Background:**

The selectivity of diagnostic analgesia of the digital flexor tendon sheath (DFTS) in horses remains debated. This study aimed to determine whether intrasynovial administration of 2% lidocaine into the DFTS increases the mechanical nociceptive threshold (MNT) of the dorsal lamellar stratum, sole, coronary band and heel bulbs, with correct placement confirmed by contrast tenography.

**Methods:**

Six healthy horses with no clinical or radiographic distal limb abnormalities were used. Forelimbs were randomly assigned, with one limb receiving 2% lidocaine and the contralateral limb receiving lactated Ringer's solution via the palmar pastern approach to the DFTS; both solutions were mixed with radiographic contrast to confirm accurate placement. MNT was measured in 10‐foot regions using a portable pressure dynamometer before and at predefined time points up to 4 hours after DFTS injection. Data were compared using Friedman and Wilcoxon tests (*p *< 0.05).

**Results:**

No significant differences in MNT were observed over time or between groups. MNT values rarely exceeded 1‐kg force, and medians in both groups showed only minor fluctuations, remaining close to 0.5‐kg force across the evaluated foot regions.

**Conclusion:**

Under the conditions of this study, contrast‐confirmed intrasynovial administration of 2% lidocaine into the DFTS did not produce detectable desensitisation of the evaluated foot regions in clinically normal horses.

## INTRODUCTION

Accurate localisation of pain is essential for the diagnosis and management of equine lameness.^1^ Although clinical examination provides valuable information, diagnostic analgesia remains one of the most important tools for identifying the anatomical source of pain and guiding subsequent imaging investigations.^2^, ^3^ The interpretation of diagnostic analgesia, however, requires a thorough understanding of the selectivity and potential limitations of each anaesthetic technique, as diffusion of local anaesthetic beyond the intended target may lead to false‐positive results and inaccurate localisation of pain.^2^

Previous studies have demonstrated that local anaesthetic agents may diffuse beyond synovial compartments after diagnostic injections, potentially affecting adjacent anatomical structures.^4^, ^5^, ^6^, ^7^, ^8^ Such diffusion may occur through the synovial membrane, along tissue planes created during needle placement, or secondary to increases in intrasynovial pressure following injection.^5^, ^6^, ^7^, ^8^ This phenomenon is of particular clinical importance during lameness investigations because improvement in lameness may be incorrectly attributed to the intended target structure, resulting in false‐positive interpretations. Consequently, unintended desensitisation may reduce diagnostic selectivity, influence the selection of advanced imaging modalities, and potentially lead to inappropriate therapeutic or surgical decisions.^2^

Diagnostic analgesia of the digital flexor tendon sheath (DFTS) is commonly used to investigate lameness associated with lesions affecting the intrasynovial portions of the superficial and deep digital flexor tendons, the manica flexoria, and inconsistent responses are seen to DFTS anaesthesia for palmar/plantar annular ligament and distal sesamoidean ligaments.^9^, ^10^, ^11^, ^12^ Although this technique is generally considered relatively selective, concerns remain regarding the possibility of unintended desensitisation of neighbouring structures following intrasynovial administration of local anaesthetic. In the distal limb, the close anatomical relationship between the DFTS and the palmar digital neurovascular bundle raises the possibility that local anaesthetic could reach sensory nerve branches supplying portions of the hoof capsule, thereby producing analgesic effects unrelated to pathology within the tendon sheath itself.^3^, ^4^ Evidence regarding the selectivity of DFTS analgesia remains conflicting. Harper et al.^13^ reported that analgesia of the DFTS did not abolish pain originating from the sole, distal interphalangeal joint or navicular bursa, supporting the selectivity of this technique for structures associated with the tendon sheath. In contrast, Jordana et al.^14^ observed distal limb desensitisation, particularly involving the heel bulbs, following DFTS analgesia performed using different injection techniques. Although Jordana et al.^14^ evaluated a larger number of injections, their study primarily addressed distal limb desensitisation associated with different approaches to the DFTS, whereas the potential effect of correctly placed intrasynovial anaesthetic on specific foot regions beyond the heel bulbs remains insufficiently characterised.

Therefore, the present study was not designed to replicate previous investigations, but to address a distinct methodological and anatomical question: whether accurately placed intrasynovial anaesthesia of the DFTS, confirmed by contrast tenography and without evidence of extravasation, can alter the mechanical nociceptive threshold of predefined foot regions. Objective assessment of the dorsal lamellar stratum, sole, coronary band and heel bulbs may help clarify whether false‐positive analgesic responses after DFTS injection can involve foot structures other than the heel bulbs. We hypothesised that accurate intrasynovial administration of 2% lidocaine into the DFTS, even in the absence of contrast‐confirmed extravasation, could induce false‐positive analgesia in selected regions of the foot. Therefore, the objective of this study was to determine whether anaesthetic infiltration of the DFTS increases the mechanical nociceptive threshold (MNT) of the dorsal lamellar stratum, sole, coronary band and heel bulbs in healthy horses.

## MATERIALS AND METHODS

### Ethics statement

Procedures performed on horses were approved by the Ethics Committee on Animal Use (CEUA) of the Federal University of Minas Gerais/UFMG (protocol CEUA 360/2017).

### Population and study design

The forelimbs of six horses—four females and two males, Mangalarga Marchador crossbred, of mean age 9.5 ± 2.4 years, weighing 388.8 ± 43.6 kg—were included. The hooves were trimmed and balanced before the experimental procedures. All horses were evaluated clinically to exclude lameness or musculoskeletal abnormalities involving the distal limbs.

The study was designed as a randomised, blinded, paired contralateral‐limb controlled experiment. For each horse, one forelimb was randomly allocated to receive 2% lidocaine, whereas the contralateral forelimb received lactated Ringer's solution as control. Treatment allocation and order of limb injection were determined before data collection by drawing sealed lots assigning treatment to the left or right forelimb of each horse. Treatments were not crossed over between limbs; therefore, no washout period was applied. Both forelimbs were injected sequentially during the same experimental session after baseline MNT measurements, and the order of limb injection followed the randomisation schedule established for each horse.

A priori sample size estimation was performed for a paired two‐treatment design with a continuous outcome, using a framework equivalent to a two‐treatment crossover calculation. The calculation was based on a one‐sided significance level of 0.05, 80% power, a minimum detectable difference between treatments of 23 units, and an assumed standard deviation of the paired differences of 18.1. Under these assumptions, six horses were estimated to provide 84% power to detect the specified treatment difference. Therefore, six horses were included in the study.

Potential bias was reduced by random allocation of treatment to limb, use of the contralateral limb as a paired control, standardisation of procedures by the same operators, and blinding of the clinician performing the injections, the investigator applying the algometer, and the evaluators assessing behavioural responses to treatment allocation wherever feasible. Details of the evaluated foot regions, injection procedure, contrast confirmation, and MNT assessment are provided below.

### Assessment sites

Foot areas were evaluated according to previous studies.^15^, ^16^, ^17^ To assess sensitivity to pressure in dorsal laminae before and after DFTS injection, a 10‐mm diameter circular section of horn was removed from the dorsal aspect of the hoof at 2 cm (L2) and at 4 cm (L4) distal to the coronary band (Figure 1). To remove the circular section of horn, horses were restrained without sedation in a stock. After scrubbing the dorsum of the hoof, a sterile, 10‐mm diameter stainless‐steel drill bit mounted to an electric screwdriver was positioned at a 90° angle to the surface of the hoof wall and advanced at low speed until resistance to drilling suddenly decreased and the remaining keratinised tissue could be depressed by pressure using a haemostat. At this point, there was no bleeding or damage to the sensitive laminae, but a foot withdrawl response could be obtained pressuring the haemostat on the hoof defect. To assess sensitivity to pressure on the sole before and after perineural injection, keratinised tissue was removed using a hoof knife until soft horn was exposed from a 1 cm^2^ area at the dorsal, lateropalmar and mediopalmar aspects of the sole (Figure 1). Keratinised tissue was removed until soft horn was exposed. At this point, there was no bleeding of solar corium, but a foot withdrawl response could be obtained pressuring a haemostat on the sole defect. Sensitivity of skin to pressure was assessed in the lateral and medial bulbs of the heels and in three regions of the coronary band: the lateral, dorsal and medial aspects (Figure 1).

**FIGURE 1:**
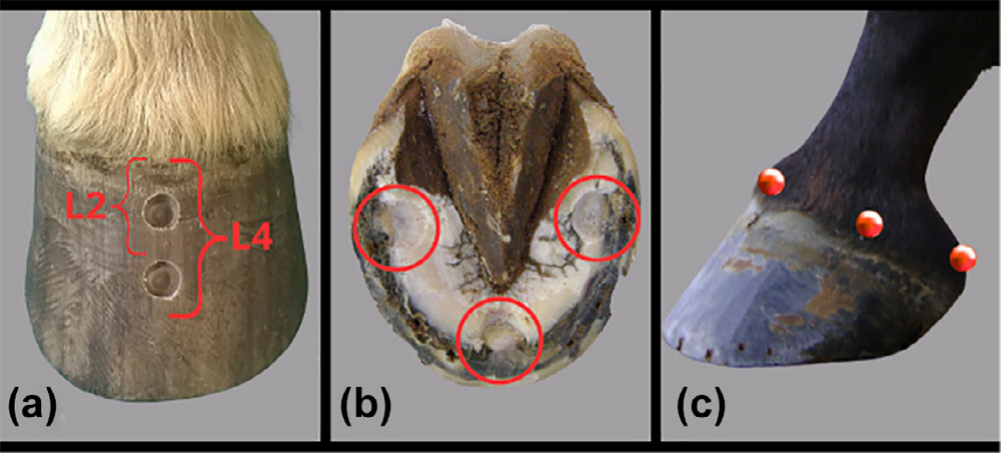
Assessment sites for sensitivity to pressure. (a) Proximal (L2) and distal (L4) dorsal laminae. (b) Dorsal, palmarolateral and palmaromedial aspects of the sole. (c) Heel bulbs and lateral, medial and dorsal aspects of the coronary band.

### Intrasynovial injection of local anaesthetic into DFTS

The horses were restrained in stocks without sedation. All DFTS injections were performed by the same clinician to minimise inter‐operator variability. The limb was maintained in a flexed, non–weight‐bearing position throughout needle placement and administration of the injectate. After surgical antisepsis of the pastern, the DFTS was injected through the distal palmar pouch approach with a 30 × 08 mm needle at 90° to the skin, between the proximal and distal digital annular ligaments and slightly lateral to the sagittal axis of the pastern. The needle penetrated the skin, subcutaneous tissue and palmar region of the DFTS.

Each DFTS was injected with either 8 mL of 2% lidocaine hydrochloride (Xylestesin 2%, Cristália Laboratory) in the treated limb or 8 mL of lactated Ringer's solution (Fresenius) in the control limb. In both limbs, 4 mL of iohexol contrast solution (Omnipaque—GE HealthCare; 300 mg iodine/mL) was added to the injectate, resulting in a total volume of 12 mL. The total injectate volume was selected based on previous studies investigating diagnostic analgesia of the DFTS and was considered sufficient to achieve adequate distribution within the synovial cavity while allowing the addition of contrast medium for radiographic confirmation of correct intrasynovial placement and exclusion of extravasation.^4^, ^11^, ^14^

Once the entire volume had been delivered, the limb was returned to the ground for radiographic assessment before subsequent MNT measurements. A lateromedial radiographic projection of the pastern region was obtained immediately after injection to confirm contrast distribution within the DFTS and to identify any leakage, extravasation or erroneous deposition of the injectate. Radiographs were obtained using a portable x‐ray machine (Poskom PXP‐20HF Plus) and a 17 × 17″ digital radiography (DR) panel (72 kV and 2.5 mAs). Synovial fluid was observed only to confirm correct needle placement; no synovial fluid sample was collected for cytological, biochemical or microbiological analysis. Successful synoviocentesis was indicated by synovial fluid within the needle hub and confirmed radiographically by contrast filling of the DFTS without evidence of peri‐synovial extravasation. If contrast radiography showed peri‐synovial extravasation or failed to confirm filling of the DFTS, data from that injection attempt were excluded, and the injection procedure was repeated in the same horse and same assigned limb/treatment after a minimum interval of 24 hours. No additional horses were enrolled. Horses were only excluded from the study if successful contrast‐confirmed intrasynovial injection could not be achieved.

### Pressure algometry

The MNT was determined using a pressure algometer with a digital handheld dynamometer.^15^, ^16^, ^17^, ^18^ Constant pressure, with a gradual increase, was applied over the evaluated region to measure the MNT up to the animal's reaction. Data were acquired in kilograms (kg) and corresponded to the minimal pressure required to induce a response, such as displacement and/or flexion of the assessed limb.^4^ During the evaluation, the horses were kept indoors, and a mask excluding the ipsilateral view of the tested limb was used to prevent stimulus prediction. Throughout the 240‐minute observation period, horses remained at rest and were not walked, trotted, lunged or actively exercised between assessment time points, except for the handling and positioning required for MNT measurements. A 1 cm^2^ flat surface tip was used for soft tissue regions, including the coronary band and heel bulbs, whereas a cone‐shaped tip was used for keratinised hoof capsule regions, including the sole and dorsal hoof wall sites corresponding to the dorsal lamellar regions located 2 and 4 cm distal to the coronary band. The MNT measurements were performed before and at 5, 10, 15, 20, 30, 60, 90, 120, 150, 180, 210 and 240 minutes after DFTS injection. Although the clinically relevant period for lidocaine‐induced desensitisation was expected to occur within the first 120 minutes after injection, extended measurements up to 240 minutes were included to characterise the complete temporal profile of foot sensitivity, identify any delayed or persistent changes in MNT, and confirm maintenance or return of baseline nociceptive responses after the expected duration of action of lidocaine. The 5‐minute post‐injection time point corresponded to the initiation of MNT assessment following completion of the injection procedure and radiographic confirmation of correct intrasynovial placement. Thereafter, measurements were obtained sequentially at all predefined foot locations using the same order of assessment for all horses and time points. Each MNT determination required approximately 5–10 seconds, including positioning of the algometer and gradual application of pressure until a behavioural response was observed. The same investigator applied the algometer throughout the study, while two blinded evaluators identified the behavioural response and confirmed the corresponding value recorded on the dynamometer. The evaluators were blinded to treatment allocation. Because limb laterality could not be concealed during handling, blinding referred to the administered treatment rather than to the physical identity of the limb. If disagreement arose between evaluators, the measurement was repeated. The measurements were performed twice, and the average values were considered.^15^, ^16^, ^17^ During each assessment period, horses were monitored for behavioural changes or signs of discomfort unrelated to the nociceptive stimulus, including agitation, repeated avoidance behaviour, persistent limb withdrawal before application of pressure, reluctance to maintain the limb in position, sweating, increased sensitivity around the injection site, or any local swelling. Any complication or behavioural change that interfered with MNT acquisition was recorded. No formal pre‐study conditioning protocol for pressure algometry was performed; however, horses were allowed to acclimatise to the stocks and handling conditions before baseline measurements. To minimise measurement variability, all foot regions were evaluated according to the same predefined anatomical sequence throughout the study. The order of limb assessment followed the randomisation schedule established for each horse; therefore, the first limb assessed was not systematically the treated or control limb. Once defined for each horse, the same limb order was maintained at all subsequent time points. The test was stopped when the applied pressure reached 6 kg to avoid tissue damage.^18^

### Statistical analysis

Considering the paired contralateral‐limb design, the repeated measurements over time, and the small number of animals included in the study, a non‐parametric statistical approach was selected. Within each group, changes in mechanical nociceptive threshold over time were evaluated using the Friedman test followed by Dunn's post hoc test. At each time point, comparisons between the lidocaine‐treated limb and the contralateral control limb were performed using the Wilcoxon signed‐rank test. Baseline MNT values were compared between groups to assess pre‐intervention comparability. Time‐course data are presented as individual values with medians in the graphical representations. All statistical analyses were performed using GraphPad Prism 7 software, with significance set at *p *< 0.05.

## RESULTS

A total of 15 DFTS injection attempts were performed in the six enrolled horses to obtain 12 contrast‐confirmed intrasynovial injections included in the final analysis. Peri‐synovial extravasation or lack of contrast‐confirmed DFTS filling was identified by contrast radiography in three of 15 injection attempts. Data from these attempts were excluded, and the injection procedure was repeated in the same horse and same assigned limb/treatment after a minimum interval of 24 hours. No additional horses were enrolled. Thus, the final dataset comprised 12 successful injections from six horses, including six lidocaine‐treated limbs and six contralateral control limbs. All successful injections and subsequent MNT assessments were completed under physical restraint without sedation. No local complications, including swelling, persistent sensitivity at the injection site, bleeding or signs of infection, were observed during the study period. No systemic adverse reactions were detected. Horses remained sufficiently compliant to allow completion of the repeated MNT assessments at all scheduled time points. Transient limb withdrawal occurred as the expected behavioural response to pressure algometry. Occasional measurements required repetition because of movement or inconsistent behavioural response; however, these events were transient and did not prevent acquisition of valid MNT values. No measurements were excluded because of lack of compliance or behavioural intolerance. Synovial fluid was observed at the needle hub in all successful injections before administration of the injectate. Before injection, limbs allocated to receive lidocaine showed numerically lower mean MNT values than limbs allocated to receive lactated Ringer's solution at all evaluated foot locations. However, baseline MNT values did not differ significantly between groups (*p *> 0.05). No significant differences in MNT were observed over time within groups or between groups at any post‐injection time point. Across all evaluated foot regions, MNT values remained low throughout the observation period in both groups. Median values showed only small fluctuations over time and remained close to baseline values, without a consistent upward trend after lidocaine administration. Individual values were generally below 1 kg force, and occasional higher values were isolated rather than sustained across consecutive time points or anatomical regions. No region showed a pattern compatible with progressive or delayed desensitisation after DFTS analgesia. Individual MNT values and medians for both groups at each time point are presented in two figures according to anatomical region: Figure 2 shows the coronary band and sole regions, whereas Figure 3 shows the heel bulbs and dorsal lamellar regions.

**FIGURE 2:**
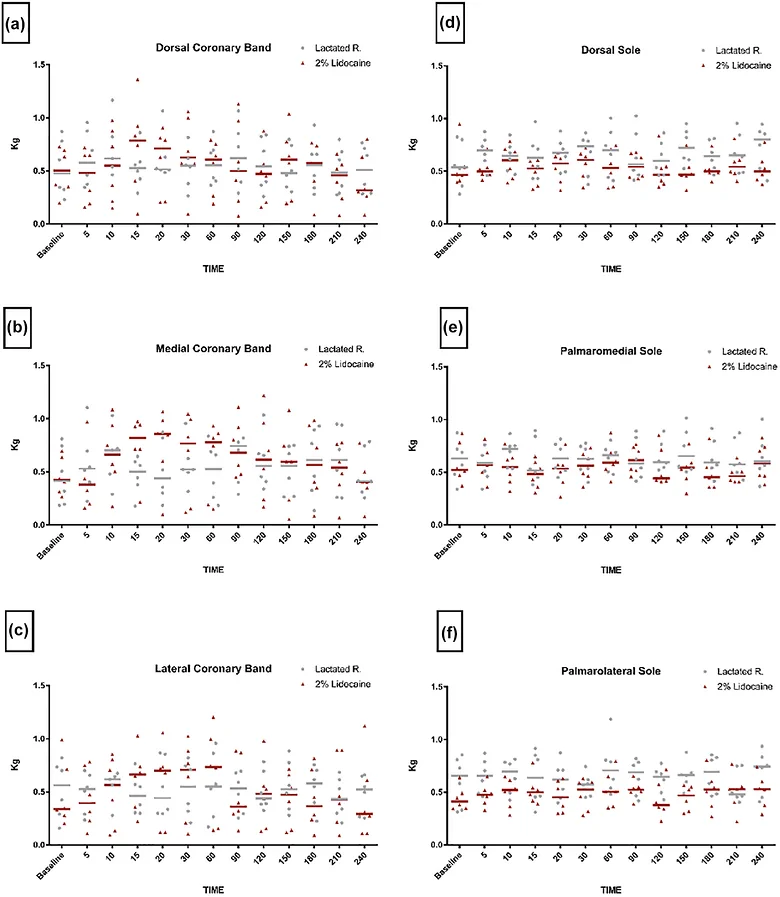
Graphical representation of mechanical nociceptive threshold values, with individual values and medians expressed in kilograms (kg), for the coronary band and sole regions of healthy equine forelimbs injected into the digital flexor tendon sheath with lactated Ringer's solution or 2% lidocaine. Panels show the coronary band: (a) dorsal, (b) medial, and (c) lateral; and sole: (d) dorsal, (e) palmaromedial, and (f) palmarolateral.

**FIGURE 3:**
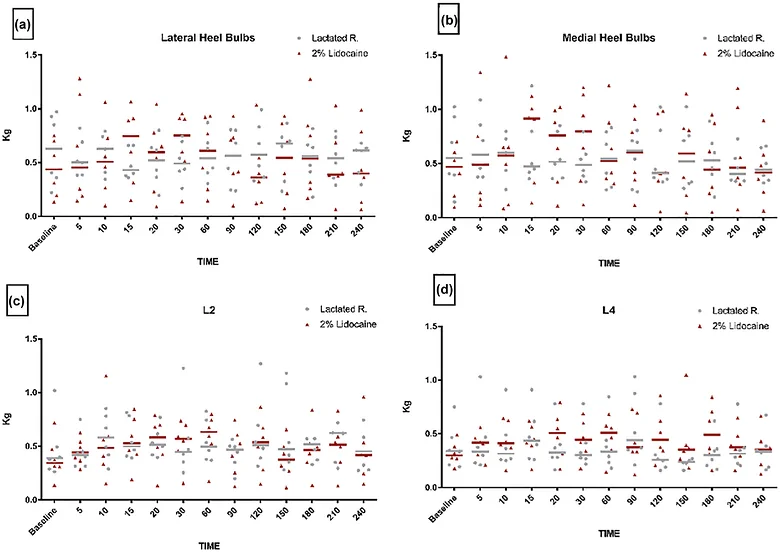
Graphical representation of mechanical nociceptive threshold values, with individual values and medians expressed in kilograms (kg), for the heel bulbs and dorsal lamellar regions of healthy equine forelimbs injected into the digital flexor tendon sheath with lactated Ringer's solution or 2% lidocaine. Panels show the heel bulbs: (a) lateral and (b) medial; and dorsal lamellar regions: (c) 2 cm and (d) 4 cm distal to the coronary band.

## DISCUSSION

The main finding of the present study was that contrast‐confirmed intrasynovial administration of 2% lidocaine into the DFTS did not increase MNT in the superficial foot regions evaluated in clinically normal horses. MNT values remained low and stable over time in both treated and control limbs, and no region showed a pattern compatible with progressive or delayed desensitisation after DFTS analgesia. Increases greater than two‐fold compared with baseline values^14^ or force tolerance ≥3 kg,^17^, ^18^ which have previously been used as indicators of foot desensitisation, were not observed. Therefore, under the controlled conditions of this study, correctly placed DFTS analgesia did not produce detectable desensitisation of the dorsal lamellar stratum, sole, coronary band or heel bulbs as assessed by pressure algometry. Clinically, these findings suggest that when DFTS analgesia is accurately performed, and no contrast evidence of extravasation is present, improvement in lameness is less likely to be explained by unintended desensitisation of these superficial sites. However, this should not be interpreted as definitive evidence of diagnostic selectivity for all sources of distal limb pain, particularly deeper anatomical structures not directly assessed by superficial MNT testing, because injection approach, local diffusion, movement, and pathological tissue changes may influence anaesthetic distribution. The clinical interpretation of the results should primarily focus on the first 120 minutes after injection, which corresponds to the expected period of clinically relevant desensitisation following lidocaine administration. Measurements obtained after 120 minutes were included to characterise the temporal profile of foot sensitivity, identify any delayed or persistent changes in MNT, and confirm maintenance of nociceptive responses after the expected duration of action of the local anaesthetic. Although limbs allocated to lidocaine had numerically lower baseline MNT values than limbs allocated to lactated Ringer's solution, these differences were not statistically significant and were considered to reflect random limb‐to‐limb variation within the paired contralateral‐limb design. Importantly, lower baseline values in lidocaine‐allocated limbs would not be expected to mask a treatment‐related increase in MNT after anaesthetic administration. The low baseline MNT values observed in this study were likely multifactorial and may be related to the high sensitivity of the evaluated distal foot regions, the use of the first clear behavioural response as the endpoint, individual variability, absence of sedation, and limited formal pre‐study conditioning to pressure algometry. As all horses were clinically sound and free of distal limb abnormalities, these low values were not interpreted as evidence of pathological hypersensitivity.

The present findings are consistent with previous studies suggesting that DFTS analgesia does not necessarily desensitise structures adjacent to the sheath or located distally within the foot. Harper et al.^13^ reported that analgesia of the DFTS did not abolish pain originating from the sole, distal interphalangeal joint or navicular bursa, and Dau et al.^19^ did not observe relevant clinical improvement after DFTS infiltration in horses with podotrochlear apparatus injuries. Similarly, relevant concentrations of mepivacaine were not detected in adjacent synovial cavities after intrasynovial administration into the DFTS.^4^ In contrast, other studies have reported findings suggesting reduced selectivity under certain conditions. Jordana et al.^14^ observed desensitisation of the heel bulbs in 29/72 limbs 15–30 minutes after injection of 2% mepivacaine into the DFTS, and positive or partially positive responses to DFTS diagnostic analgesia have been reported in horses with distal sesamoidean ligament injuries.^20^, ^21^ These apparently conflicting findings suggest that the effects of DFTS analgesia may depend on injection technique, anatomical site assessed, presence of pathology, local diffusion and method used to assess desensitisation. The present study addressed a distinct question by combining contrast confirmation of intrasynovial placement without extravasation with repeated quantitative assessment of predefined foot regions beyond the heel bulbs.

The technique used for DFTS synoviocentesis may influence the likelihood of unintended diffusion or false‐positive analgesic responses. Several techniques for DFTS injection have been described,^22^ and the proximal approach has been associated with a higher leakage score and closer proximity to the lateral palmar nerves.^23^ Peri‐synovial leakage of local anaesthetic or proximity of the injectate to adjacent sensory nerves could contribute to desensitisation of structures outside the DFTS. For this reason, the distal palmar pouch approach was selected in the present study. Contrast tenography also provided an important methodological control, because only contrast‐confirmed intrasynovial injections without evidence of extravasation were retained for MNT analysis. This is clinically relevant because combined contrast tenography and intrathecal diagnostic analgesia are used during investigation of suspected DFTS pathology, allowing assessment of contrast distribution while also evaluating the analgesic response.^11^, ^24^ In this context, confirming correct intrasynovial distribution and identifying peri‐synovial extravasation are important for interpretation of both experimental MNT findings and clinical lameness responses. Excluding injection attempts with peri‐synovial extravasation or lack of contrast‐confirmed DFTS filling reduced the risk of attributing changes in foot sensitivity to DFTS analgesia, when they could instead have resulted from inadvertent peri‐synovial deposition or diffusion of the injectate to adjacent soft tissues or nerves.

These results should be interpreted in light of several limitations. First, the effectiveness of lidocaine within the DFTS was not directly assessed using a positive control or a clinical model of DFTS‐related pain. Because the horses were clinically normal and did not have lameness or painful DFTS pathology, it was not possible to determine whether intrasynovial lidocaine would have abolished pain arising from the tendon sheath itself. Contrast tenography confirmed correct intrasynovial deposition and absence of extravasation, but it did not directly confirm the pharmacological effectiveness of the local anaesthetic. Therefore, the findings should be interpreted specifically as evidence that contrast‐confirmed DFTS injection with 2% lidocaine did not produce detectable false‐positive analgesia in the evaluated superficial foot regions, rather than as a direct assessment of the clinical efficacy of DFTS analgesia for painful tendon sheath lesions. Second, this experimental study included a limited number of horses, which limits statistical power and the ability to detect subtle treatment effects. Third, the injectate volume and anaesthetic agent used in the present study may not fully represent all clinical block‐tenogram protocols. A total volume of 12 mL was used, whereas larger volumes, such as 20 mL, may be used in some combined intrathecal diagnostic analgesia and contrast tenography procedures. In addition, lidocaine was used in the present study, whereas mepivacaine is commonly used for diagnostic analgesia in horses. Differences in volume, anaesthetic agent, and tissue diffusion characteristics may influence the degree and distribution of desensitisation. Fourth, only the distal palmar pouch approach was evaluated, and the findings should not be extrapolated directly to other DFTS injection approaches that may differ in leakage risk or proximity to adjacent sensory nerves. Fifth, horses remained at rest during the 240‐minute evaluation period and were not actively exercised between assessment time points. Movement may influence intrasynovial pressure, local anaesthetic distribution, peri‐synovial diffusion, and the clinical expression of lameness after diagnostic analgesia. Finally, the study primarily assessed superficial external foot regions accessible to pressure algometry, whereas many clinically important causes of distal limb lameness originate from deeper internal sensitive structures. Thus, the absence of increased superficial MNT should not be interpreted as evidence that DFTS analgesia cannot affect deeper pain‐generating structures in clinical cases.

Overall, the present study suggests that correctly placed DFTS analgesia, confirmed by contrast tenography and performed without extravasation, does not produce measurable desensitisation of the superficial foot regions evaluated in resting, clinically normal horses. Further studies including larger populations, dynamic post‐injection assessment, positive controls, different injectate volumes, alternative local anaesthetic agents, other DFTS injection approaches, and horses with naturally occurring lesions involving structures within or adjacent to the DFTS are warranted to further clarify the selectivity and clinical interpretation of DFTS diagnostic analgesia.

## CONCLUSIONS

In clinically normal horses at rest, contrast‐confirmed intrasynovial administration of 2% lidocaine into the DFTS did not alter the mechanical nociceptive thresholds of the dorsal lamellar stratum, sole, coronary band or heel bulbs. These findings suggest that when correctly placed and without evidence of extravasation, DFTS diagnostic analgesia does not produce detectable desensitisation of the evaluated foot regions. Further studies in horses with naturally occurring DFTS‐related pain and dynamic post‐injection assessment are warranted to confirm the clinical interpretation of these findings.

## AUTHOR CONTRIBUTIONS

**Letícia de Oliveira Cota, Bruno Dondoni Malacarne, Cahuê Francisco Rosa Paz, Armando de Mattos Carvalho** and **Andressa Batista da Silveira**: Contributed to the conception and design of the study, definition of the research objective, data analysis and interpretation, drafting of the manuscript, critical revision for important intellectual content, and approval of the final version to be published. **Lucas Antunes Dias, Antônio Catunda Pinho Neto, Mayara Gonçalves Correa, Marina Alcantara Cavalcante, Marina de Souza Luz da Cunha** and **Camila Ferreira Rodrigues e Silva**: Contributed to the investigation, methodology, acquisition of data, data curation, interpretation of the results, critical revision of the manuscript for important intellectual content, and approval of the final version to be published.

## FUNDING INFORMATION

The authors received no specific funding for this work.

## ETHICS STATEMENT

Procedures performed on horses were approved by the Ethics Committee on Animal Use (CEUA) of the Federal University of Minas Gerais/UFMG (protocol CEUA 360/2017).

## CONFLICTS OF INTEREST

The authors declare they have no conflicts of interest.

## Data Availability

The data supporting the findings of this study are available from the corresponding author upon reasonable request.
